# Synthesis and biological evaluation of thiazolidinedione derivatives with high ligand efficiency to *P. aeruginosa* PhzS

**DOI:** 10.1080/14756366.2021.1931165

**Published:** 2021-06-03

**Authors:** Thamires Quadros Froes, Bianca Trindade Chaves, Marina Sena Mendes, Rafael Matos Ximenes, Ivanildo Mangueira da Silva, Priscila Brandão Gomes da Silva, Julianna Ferreira Cavalcanti de Albuquerque, Marcelo Santos Castilho

**Affiliations:** a Programa de Pós-graduação em biotecnologia da, Universidade Estadual de Feira de Santana, Feira de Santana, Brazil; b Faculdade de Farmácia da, Universidade Federal da Bahia, Salvador, Brazil; c Departamento de Antibióticos da, Universidade Federal de Pernambuco. Av. Prof. Moraes Rego, Recife-Pe, Brazil; d Programa de Pós-Graduação em Farmácia da, Universidade Federal da Bahia, Salvador, Brazil

**Keywords:** Thiazolidinone, pyocyanin biosynthesis inhibition, *P. aeruginosa*, antimicrobial resistance

## Abstract

The thiazolidinone ring is found in compounds that have widespan biology activity and there is mechanism-based evidence that compounds bearing this moiety inhibit *P. aeruginosa* PhzS (*Pa*PzhS), a key enzyme in the biosynthesis of the virulence factor named pyocyanin. Ten novel thiazolidinone derivatives were synthesised and screened against *Pa*PhzS, using two orthogonal assays. The biological results provided by these and 28 other compounds, whose synthesis had been described, suggest that the dihydroquinazoline ring, found in the previous hit (**A**- Kd = 18 µM and LE = 0.20), is not required for *Pa*PzhS inhibition, but unsubstituted nitrogen at the thiazolidinone ring is. The molecular simplification approach, pursued in this work, afforded an optimised lead compound (**13**- 5-(2,4-dimethoxyphenyl)thiazolidine-2,4-dione) with 10-fold improvement in affinity (Kd= 1.68 µM) and more than 100% increase in LE (0.45), which follows the same inhibition mode as the original hit compound (competitive to NADH).

**Executive summary**
- PhzS is a key enzyme in the pyocyanin biosynthesis pathway in *P. aeruginosa.*
- Orthogonal assays (TSA and FITC) show that fragment-like thiazolidinedione derivatives bind to *Pa*PhzS with one-digit micromolar affinity.
- Fragment-like thiazolidinedione derivatives bind to the cofactor (NADH) binding site in *Pa*PhzS.
- The molecular simplification optimised the ligand efficiency and affinity of the lead compound.

## Introduction

**Figure 1.**
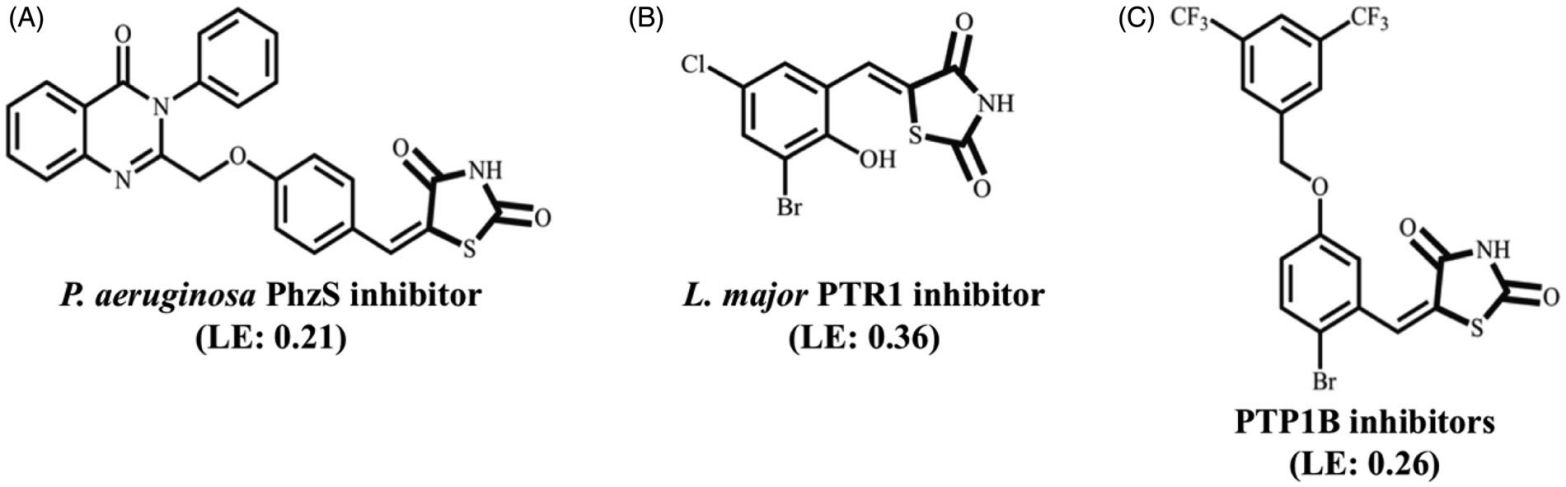
Biological activity of selected 4-thiazolidinone derivatives.

Compounds bearing the 4-thiazolidinone, 2,4-thiazolidinedione, rhodanine (2-thioxo-4-thiazolidinone) moieties have been explored by medicinal chemists since the 1960s^1^^,^^2^. In fact, many authors claim that 4-thiazolidinone ring might be considered a privileged scaffold for drug design efforts^3^^,^^4^. One of the main achievements that have fuelled the drug design efforts with 4-thiazolidinone derivatives was the introduction of antidiabetic drugs to the market (i.e. peroxisome proliferator-activated receptor-g (PPAR) agonists^5^ and aldose reductase inhibitors^6^) However, this class of compounds has also shown promise in several fields (Figure 1). For instance, 5-arylidene-2,4-thiazolidinone derivatives are low micromolar inhibitors of Pteridine reductase 1 from *Leishmania major*^7^ that are also active against *L. braziliensis* and *L. infantum* promastigotes^8^, and benzylidene-2,4-thiazolidinedione derivatives^9^ and thiazolidine-2,4-diones derivatives with a carboxylic ester substituent at N-3^10^ have shown activity against protein-tyrosine phosphatase 1B (PTP1B). More recently, Froes and co-workers included the inhibition of *P. aeruginosa* PhzS to the list of biological activities ascribed to compounds bearing the 2,4-thiazolidinedione ring and underscored that these compounds might be employed to battle resistant bacteria^11^.

Antimicrobial resistance (AMR) causes more than 700,000 deaths each year^12^ and burdens approximately 2 million patients in the USA^13^. To make matters worse, these figures may escalate to 10 million deaths per year if no action is taken^14^. Nevertheless, the investment in the development of novel antibiotics has been steadily decreasing^15^^,^^16^. The roots for this problem have already been discussed, but most of them are somehow related to the fact that both bactericidal and bacteriostatic drugs are responsible for an evolutionary pressure that selects resistant strains^17^. In order to overcome this dilemma, antivirulence drugs, which aim at ‘disarming’ the pathogens instead of killing them, have been pursued^18–21^. In contrast to previous studies that focus on quorum sensing modulation^22–25^, in this work we target enzymes from the pyocyanin (PYO) biosynthesis pathway in *P. aeruginosa*, a Gram-negative bacillus with high degree of resistance to available drugs^26^, in particular PhzS, an enzyme that catalyses the last step of the PYO biosynthesis, because A) genetic validation (*phzs* gene knockout) suggests that this protein is crucial for *P. aeruginosa* infection progression^27^; B) *in silico* tools predict this target to be druggable^28^; C) the reaction catalysed by PhzS is NADH-dependent^29^^,^^30^. This last feature prompted the analysis of thiazolidine-2,4-dione derivatives that might mimic the interaction profile of the purine ring from NADH. These efforts already led to the identification of (E)-5–(4-((4-oxo-3-phenyl-3,4-dihydroquinazolin-2-yl)methoxy)benzylidene)thiazolidine-2,4-dione (**A**) as a low micromolar affinity (Kd= 18 µM) inhibitor of PhzS from *P. aeruginosa*^11^. If one compares the ligand efficiency (LE) of this compound (0.21) to the LE of other thiazolidine-2,4-dione derivatives that display a similar competitive mechanism to NADPH (i.e. **B**- LE = 0.36), it becomes clear that the initial hit should have poor complementarity to its binding site. In order to identify the moieties that are essential for PhzS inhibition, molecular modification strategies, such as replacement of dihydroquinazoline moiety by lipophilic, less-bulky substituents, the addition of aromatic rings at the nitrogen from the thiazolidine-2,4-dione ring, etc. were carried out. The biological profile of those compounds shows that dihydroquinazoline moiety is detrimental to LE and that N-substitution is not compatible with *Pa*PhzS binding. Moreover, the most promising lead compound reported in this work not only has improved affinity to its target (Kd= 1.68 µM) and increased LE (0.45) but also follows the same mode of inhibition of the previous hit (competition with NAD+).

## Materials and methods

The reagents were purchased from Sigma-Aldrich and the solvents from the Vetec and Dinâmica brands. The reactions were monitored by thin-layer chromatography (TLC) using Merck Silica gel 60 F254 chromatographic plates, 0.25 mm thick, observed under ultraviolet light of two different wavelengths (254 or 366 nm).

The reactions were carried out in an oil bath with Fisatom model 752 A hot plate with magnetic stirring. The solvents were evaporated under reduced pressure using a Fisatom rotary evaporator, model 550. The melting points were measured in capillaries using a Quimis fusiometer, model 340.23, and are uncorrected. The products were weighed on a Bel analytical balance, model Mark 210 A and dried in a Liobras freeze dryer, L101.

All compounds were characterised by NMR ^1^H, NMR ^13^C, and DEPT analyzes. The NMR ^1^H and NMR ^13^C spectra were obtained on Varian instruments, model Unity Plus (400 MHz for ^1^H; 100 MHz for ^13^C) or Bruker AMX (300 MHz for 1H and 75.5 MHz for ^13^C), using tetramethylsilane as an internal standard. The multiplicity of signals in the NMR ^1^H spectra were designated as follows: s (singlet); d (doublet); t (triplet); dd (double doublet); q (quartet); or m (multiplet). The IR spectra were obtained using an Infra-red Absorption Spectrophotometer – FTIR Bruker Model IFS 66 or Perkin Elmer – Spectrum 400 using KBr tablets. For high-resolution mass spectra (HRMS), the electrospray (ESI) ionisation technique was used in positive (ESI^+^) or negative (ESI^-^) modes with time-of-flight (TOF) detection, measured on a Shimadzu LC-ESI-qTOF-MS device.

### Chemistry

**Figure 2.**
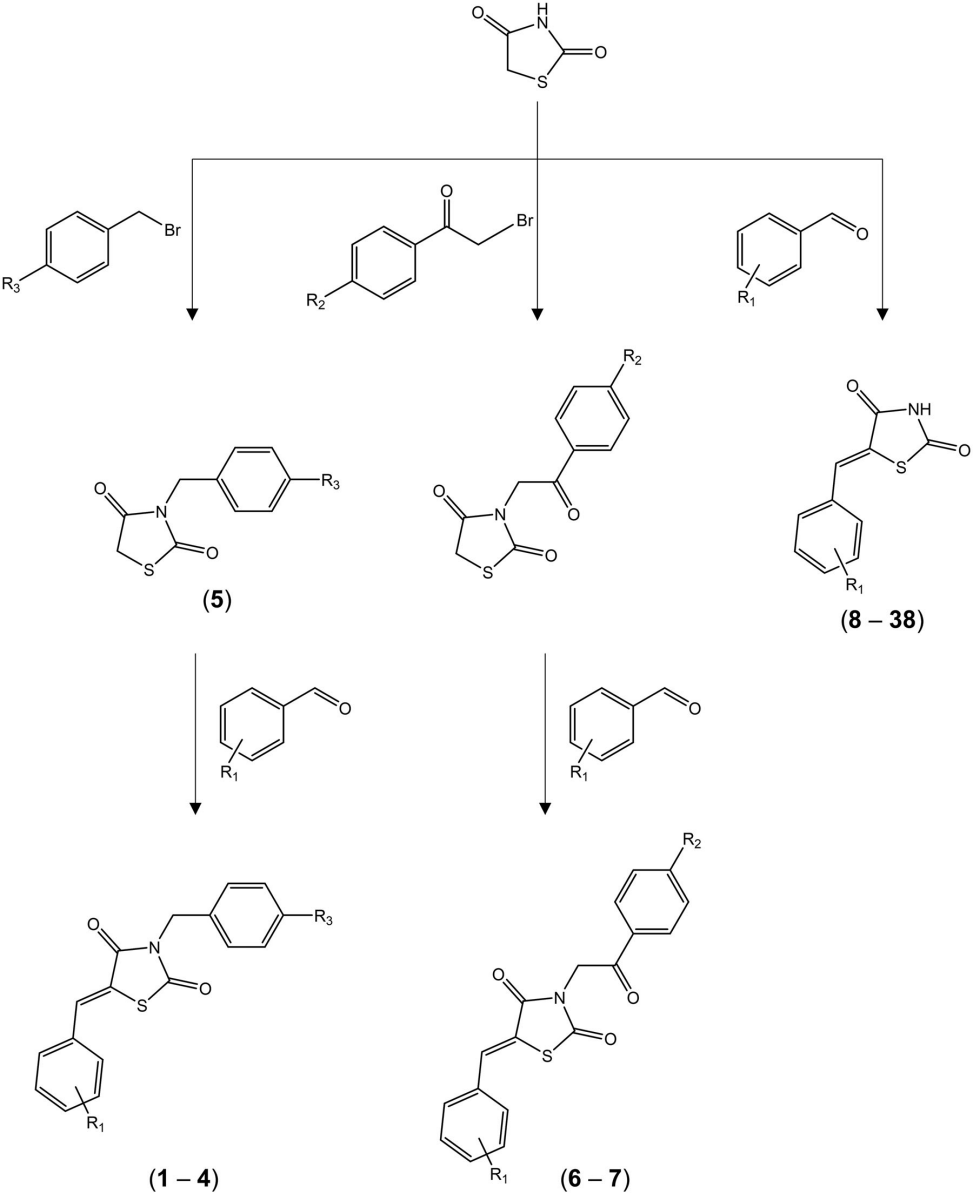
General synthesis steps to obtain 2,4-thiazolidinedione derivatives.

Thiazolidine-2,4-dione derivatives (**1 − 38 -** Tables 1 and 2) were obtained by well-established synthetic routes based on Knoevenagel condensation. Compounds **2**^31^**, 4**^32^**, 5**^33^^,^^34^**, 8–9**^7^**, 10**^35^**, 11–17**^7^**, 18**^36^**, 19**^8^**, 22**^37^^,^^38^**, 23**^39^**, 24**^35^^,^^40^**, 25**^37^^,^^41^^,^^42^**, 26**^43^**, 27**^43^^,^^44^**, 29**^45^**, 30**^46^**, 31**^3^^,^^47^**, 32**^48^**, 33**^8^**, 35**^36^**, 37**^33^^,^^49^ (Supplementary material) were synthesised according to previously published protocols, whereas novel compounds were synthesised through the general steps described in Figure 2.

**Table 1.** N-substituted 5-arylidene-thiazolidine-2,4-dione derivatives assayed against *Pa*PhzS.

*Novel compounds.

**Table 2.** 5-arylidene-thiazolidine-2,4-dione derivatives assayed against *Pa*PhzS.

*Novel compounds.

The starting compound (thiazolidine-2,4-dione) was obtained as described by Libermann and Himbert (1948)^50^, with the modifications proposed by Albuquerque et al. (1995)^51^. This reaction occurs by condensation of monochloroacetic acid and thiourea in an aqueous medium under reflux for 24 h. Molecular formula C_3_H_3_O_2_NS; yield 78%; mp 118–120 °C; *R*_f_ 0.48 (0.9:0.1 CHCl_3_/MeOH). Recrystallization: water.

#### General synthesis of novel N-substituted 5-benzylidene-thiazolidine-2,4-dione derivatives (1, 3, 6 and 7)

Compounds **1**, **3**, **6** and **7** were synthesised in two steps. First, a solution of KOH dissolved in methanol was added drop-wise, under constant stirring, to thiazolidine-2,4-dione solubilised in 10 ml of methanol (1:1 molar ratio). After 15 min, under magnetic stirring, the appropriate phenacyl bromide was added drop-wise to the reaction mixture, which was kept under reflux for 24 h. After that, the reaction product was filtered and washed with ice-cold ethanol. In the second step, the product obtained in the previous step was dissolved in methanol and two drops of piperidine. After 10 min, equimolar amounts of the appropriate aromatic benzaldehyde were added to the flask, which was kept at 75 °C, under magnetic stirring and reflux for 8 h. Then, the product was filtered and washed with ice-cold ethanol, crystallised, and dried in an oven at 40 °C.

##### 1. (Z) 5–(5-chloro-2-hydroxybenzylidene)-3-(3chlorobenzyl)thiazolidine-2,4-dione

Chemical formula: C_17_H_11_Cl_2_NO_3_S; MW 378.9837; *mz* 378.9837 (100%); Yield: 55%; MP 201–202 °C; *R*_f_. 0.55 (CHCl_3_:MeOH; 9.6:0.4). Infra-red (KBr, cm^−1^ 1716 (C=O_4_); 1676 (C=O_2_); 1605 (C=C). NMR ^1^H (300 MHz, CDCl_3_ (*δ*ppm): 5.51 s 2H, CH_2._ 8.21; 5.36 (s 1H OH); 8.22 s 1H C=H; 6.83 (d 1H *J* = 7.5) 7.19 (d 1H *J* = 7.45) 7.14 (s 1H Ar) Benzylidene. 7.44 (s 1H Ar); 7.29 (ddd 1H, H4 *J* = 7.10; 1.5;1.5) Ar; 7.12 (ddd *J* = 7.5, 1.5, 1.5 Ar), benzyl. NMR ^13^C (CDCl_3_) (DEPT MHz):*δ* ppm: 171.51 (C=O_2_); 163.3 (C=O_4_) 116.00 C=C; 143.79 C=H, 47.1 CH_2_; 144.58; 135 01; 133.12; 127.21; 126.11; 132.21 (benzyl). 154.3, 131.2;128.2; 127.1;119.1; 118.1 (benzylidene) HRMS^+^, 378.9837 (100%) calculated, 378.9828.

##### 3. (Z)-3–(3-chlorobenzyl)-5–(2,6-dichlorobenzylidene)thiazolidine-2,4-dione

Chemical formula: C_17_H_10_Cl_3_NO_2_S; MW 396,9498; *mz* 396.9498 (100%); Yield: 57%; MP 110–111 °C; *R*_f_. 0.47 (Hex:CHCl_3_, 0.7:0.3). Infra-red (KBr, cm^−1^): (C=C) 1743 (C=O_4_), 1673 (C=O_2_); 1610 (C=C). NMR ^1^H (300 MHz, CDCl_3_ (*δ*ppm): 5.56 (s 2H (CH_2_); 8.21 s 1H; CH=); 7.52 (dd H_3_–H_5_ *J* = 7.5, 1.5) benzylidene); 7.12 (ddd H_3_–H_6_ *J* = 7.57, 1.50, 1.45). 7.28) (dd 1H, *J* = 7.51, 1.48 Ar); 7.30 (dd 1H, *J* = 7.50, 1.50) Ar), benzyl. NMR ^13^C (CDCl_3_) (DEPT (75.4 MHz): *δ*ppm: 172.9.1. (C=O_2_); 163.7.21 (C=O_4_) 115.41 C=C; 146.81 C=H; (143.13; 131.8; 131.21; 133.3; 135.36; 126.65; benzyl). 136.73; 131.63; 129.87; 126.55; 127.53; 132.73, benzylidene). HRMS^+^ 396. 9498 (100%) calculated, 396.8989 found.

##### 6. (Z)-3–(2-(4-methoxyphenyl)-2-oxoethyl)-5–(4-methylbenzylidene)thiazolidine-2,4-dione

Chemical formula: C_20_H_17_NO_4_S; MW. 367.0878; *mz* 367.0878 (100%) Yield: 78%; MP 174–176 °C; *R*_f_ 0.49 (Hex:EtOAc, 0.52:0.48). Infra-red (KBr, cm^−1^ 1746 (C=O_4_), 1676 (C=O_2_); 1610 (C=C). NMR ^1^H (300 MHz, CDCl_3_ 300 MHz (*δ*ppm): 7.96 (s 1H, CH=); 6.13 s 2 H (CH_2_). 3.85 (s 3H, OCH_3_); 2.37 s 3H (CH_3_); 7.61 (d H_2_–H_6_ J = 7.30, 1.5 Ar); 7.20 (d H_3_–H_5_ (*J* = 7.26, 1.5) Ar; (benzylidene). 7.87 (d H_2_–H_6_ (*J* = 7.26, 1.5 Ar) (benzyl). NMR ^13^C (CDCl_3_) (DEPT (75.4 MHz):*δ* ppm: 55.96, CH_3_; 55.71 CH_2_; 197.12 C=O; 174.73 (C=O); 167.3 C=O; 117 C=CH; 145.71 C=CH; 21.45 CH_3_; 168.09; 114.33 × 2; 132.3 × 2; 129.9 (Ar); 134.8; 129.3 × 2; 128.7 × 2; 138.9 Ar. HRMS^+^, (367.0878 (100%) calculated, 367.0873 (100%) found.

##### 7. (Z)-5–(3,4-dichlorobenzylidene)-3–(2-(4-nitrophenyl)thiazolidine-2,4-dione

Chemical formula: C_18_H_10_Cl_2_N_2_O_5_S; MW. 367.4183; *mz* 367.0878 (100%); Yield: 78%; MP 230–231 °C; *R*_f_ 0.49 (CHCl_3_:Hex, 0.8:0.2). Infrared (KBr, cm^−1^ 1749 (C=O_4_), 1678 (C=O_2_); 1615 (C=C). NMR ^1^H (300 MHz, CDCl_3_ 300 MHz (*δ*ppm): 5.88 (s 2H CH_2_); 8.33 (d 1H *J* = 7.12), 8.38 (d 1H, *J* = 7.15); 7.97s 1H C=H; 7.57 (d 1H *J* = 7.52 Ar); 7.41 d 1H *J* = 7.51 Ar) 7.22 (s 1H Ar). NMR ^13^C (CDCl_3_) (DEPT (75.4 MHz):*δ* ppm: 55.15 (CH_2_), 194.7 (C=O). 171.81 (C=O_2_); 163.33 (C=O_4_); 115.61 C=C; (127.98, 121.7, 153.02, 127.98, 121.7, 140.87 Ar) (133.61; 126.7; 128.41; 131.81; 133.15 Ar). HRMS^+^, 367.0878 calculated, 367.0715 found.

#### General synthetic steps for novel 5-arylidene-thiazolidine-2,4-dione derivatives (7, 19–21, 28, 33–34, 36, 38)

Method: In a round bottom flask, 0.100 g (0.85 mmol) of Thiazolidine-2,4-dione (Ju-32) and 4 ml of ethanol were mixed, with stirring, until the complete solubilisation. Then, 2 drops of piperidine were added. The mixture was kept under magnetic stirring at room temperature for 10 min. After this time, 0.17 g (0.85 mmol) of benzaldehyde was added. The flask was kept at 75 °C, under magnetic stirring and reflux for 5–15 h, depending on each aldehyde. After cooling the solvent, the product was filtered through ice-cold ethanol.

##### 20. (Z) 5–(2-bromo-3-hydroxy-4-methoxybenzylidene)thiazolidine-2,4-dione

Chemical formula: C_11_H_8_BrNO_4_S; MW 328.9357; *mz* 328.9357 (100%). Yield: 59%; MP 245 °C *R*_f_. 0.51 (0.9:0.1 (CHCl_3_: MeOH). Infra-red (KBr, cm^−1^): 1746 (C=O_4_), 1676 (C=O_2_); 1610 (C=C). NMR ^1^H (300 MHz, CDCl_3_ 300 MHz (*δ* ppm): 13.12 (s 1H (NH); 8.21 (s 1H, CH=); 6.73 (d 1H, H_5,_ *J* = 7,48); 3.81 (s 3H, CH_3_) 5.37 (s 1H, OH). NMR ^13^C (CDCl_3_) (DEPT (75.4 MHz):*δ* ppm: 167.5. (C=O_2_); 166.27 (C=O_4_); 117.01 C=C; 142.58 CH=; (Ar 130.01; 115.31; 142.15; 153.18; 111.32; 123.01). HRMS^+^, (328.9357 (100%) calculated, 328.9348 (100%) found.

##### 21. (Z)-5–(3-bromo-4-methylbenzylidene)thiazolidine-2,4-dione

Chemical formula: C_11_H_8_BrNO_2_S; MW 296.9459; *mz* 296.9459 (100%); Yield: 61%; MP 196–197 °C. *R*_f_ 0.50 (8.5:1.5 Hex. AcOEt). Infra-red (KBr, cm^−1^): 1745 (C=O), 1673 (C=O); 1610 (C=C). NMR ^1^H (300 MHz, DMSO-d6): (*δ* ppm) 12.89 NH (amide), 7.94, s 1 H CH=; 7.47 s 1 H_2_; 7.10, d 1H_5_ *J* = 7.43 Ar) 7.55 dd 1H_6_ *J* = 7.5, 1.48; 2.37 s 3H (CH_3_). NMR ^13^C (DEPT (75.5 MHz, DMSO-d6): (*δ* ppm) 166.92 (C=O); 167.0 (C=O); 115.98 C=C; 143.23 CH=; (133.97, 129.87, 124.11; 137.17; 130.81; 128.01 Ar); 23.91 (CH_3_). HRMS^+^, 296.9459 (100%) calculated 296.9145 found.

##### 28. (Z-5–(2,4,5-trimethoxybenzylidene)thiazolidine-2,4-dione)

Chemical formula: C_13_H_13_NO_5_S; MW 295.0419; *mz* 295.0514 (100%); Yield: 67%; MP. 158–160 °C *R*_f_. 0.52 (0.83:0.17 Hex; AcOEt). Infra-red (KBr, cm ^−1^): 1747 (C=O_4_), 1651 (C=O_2_); 1611 (C=C). NMR ^1^H (300 MHz, CDCl_3_ 300 MHz (*δ* ppm): 12.76 (s 1H NH); 8.21 (s 1H, CH=); 3.84 (s 3 OCH_3_. orto, meta, para Ar) 6.43 (s 1H CH meta) 6.70 (s 1H CH para). NMR ^13^C (CDCl_3_) (DEPT (75.4 MHz): *δ* ppm: 166.9 (C=O_2_); 166.35 (C=O_4_) 116.31 C=C; 143.57 CH=; 55.91 × 3 CH_3_. 108.5; 136.1; 99.31; 151.19; 142.51; 111.02 CH, Ar). HRMS^+^, 295.0514 (100%) calculated, 295.0413 found.

##### 34. (Z)-5-(isoquinolin-4-ylmethylene)thiazolidin-2,4-dione

Chemical formula: C_13_H_8_N_2_O_2_S; MW 256.2798; *mz* 256.0306 (100%); Yield: 77%; MP 118–119.5 °C. *R*_f_. 0.51 (0.99:0.01 MeOH:CHCl_3_). Infra-red (KBr, cm ^−1^):), 1623 (C=C), NMR ^1^H (300 MHz, CDCl_3_ 300 MHz (*δ* ppm): 12.32 (s 1H (NH); 11.12 s 1H NH; 7.97; (s 1H, CH=); 7.87 (s 1 H, CH); 7.15; (d 1H)d Ar); 7.15 (dd, 1H (*J* = 7.1; 1.5)) Ar; 6.98 (dd 1H, *J* = 7.51; 1.5). 7.27 (dd 1H *J* = 7.1; 1.5). 7.68 dd *J* = 7.51. 1.50 Ar). NMR ^13^C (CDCl_3_) (DEPT (75.4 MHz):*δ* ppm: 166.81 (C=O_2_); 165.27 (C=O_4_) 121.11 C=C; 142.8 CH=; (137.26; 112.8; 121.93; 120.03; 120.73; 119.87; 128.07 Ar) HRMS^+^, 256.2798 (100%) calculated, 256.0235 found.

##### 36. (Z)-5-((2-hydroxynaphthalen-1-yl)methylene)thiazolidine-2,4-dione

Chemical formula: C_14_H_9_NO_3_S; MW 271.0303; *mz* 271.0303. (100%); Yield: 69%; MP 194–195 °C; *R*_f_. 0.49 (0.96:0.04, CHCl_3_:MeOH). Infra-red (KBr, cm^−1^): 1754 (C=O_4_), 1676 (C=O_2_); 1615 (C=C). NMR ^1^H (300 MHz, CDCl_3_ 300 MHz (*δ* ppm): 12.32 (s 1H (NH); 8.21 (s 1H, CH=); 5.37 (s 1H, OH); 7.78 (dd CH *J* = 7.1, 1.5); 7.52 (dd, H_1;_ H_3_) *J* = 7.59; 1.5). 7.43 (dd *J* = 7.2, 1.5). 7.42 (dd *J* = 7.4, 1.51); 7.42, (dd *J* = 7.4, 1.51); 8.01 dd (*J* = 7.1; 1.5). 7.76 (d 1H, (*J* = 7.1); 6.97 (d 1H, *J* = 7.0). NMR ^13^C (CDCl_3_) (DEPT (75.4 MHz): *δ* ppm: 167.51 (C=O_2_); 166.38 (C=O_4_) 117.06 C=C; 143.79 CH=; 155.3 C–OH; 120.0; 130.0 128.4; 130.1; 127.5, 123.5; 127.0; 122.8 128.7 Ar.). HRMS^+^, 271.0303 (100%). Calculated, 271.0299 found.

##### 38. (Z)-5-((E)-3–(2-nitrophenyl)allylidene)thiazolidine-2,4-dione

Chemical formula: C_12_H_8_N_2_O_4_S; MW 276.2679. *mz* 276.0205. (100%); Yield: 69%; MP 119–120 °C. *R*_f_. 0.52 (0.86:0.14 Hex; AcOEt). Infra-red (KBr, cm^−1^): 1746 (C=O_4_), 1677 (C=O_2_); 1612 (C=C). NMR ^1^H (300 MHz, CDCl_3_ (*δ* ppm): 13.24 (s 1H (NH); 7.97 (s 1H, CH=); 6.89 (d 1H, CH=) 7.39 (d 1H CH=) (8.01 dd 1H, *J* = 7.5, *J* = 1.5, Ar) (7.82 dd *J* = 7.5; *J* = 1.5), 7.91 (ddd 1H *J* = 7.5, 1.5, Ar); 7.89 (dd; 2H *J* = 7.0; 1.5 Ar). NMR ^13^C (CDCl_3_) (DEPT (75.4 MHz): *δ* ppm: 168.12 (C=O_2_); 167.91 (C=O_4_) 119.07 C=C; 136; 126.04, 47.9; 124.01; 123.90; 129.01; 135.60; 127.28 HRMS^+^, 276.0205 (100%) calculated, 276.0201 found.

### Biological methods

#### Expression and purification enzymes

The expression and purification of the PhzS from *P. aeruginosa* was performed as described by Greenhagen and co-workers^29^ with minor modifications to improve the overall yield^11^. Briefly, *E. coli* Rosetta (DE3) cells, containing the pET28a plasmid that codes for PhzS from *P. aerugi*nosa, were grown at 37 °C (180 RPM), in LB medium supplemented with kanamycin (30 µg/mL) and chloramphenicol (34 µg/mL), until the OD600 reached 0.6−0.8. Then, Isopropyl-b-D-thiogalactoside (IPTG) was added to the culture (final concentration 250 µM) and the temperature was reduced to 18 °C. After 24 h, the cells were harvested by centrifugation (100,000 RPM, Avanti J-E centrifuge (Beckman Coulter), 4 °C, 30 min) and resuspended in Potassium Phosphate buffer (50 mM) pH 8.0, supplemented with 300 mM NaCl and 1 mM phenylmethanesulphonylfluoride (PMSF). Next, the cells were disrupted by sonication (10–15 s bursts with 30 s intervals between each burst, 8 Watts) in an ice bath. The soluble fraction was clarified by centrifugation (16000 rpm, 20 min, 4 °C) and then loaded onto a HisTrap HP column (GE Healthcare), pre-equilibrated with buffer A (Potassium Phosphate 50 mM (pH 8.0), NaCl (300 mM) and imidazole (20 mM)). The column was washed with 20 column volumes (CV) of buffer A and then with increasing concentrations of imidazole (50–500 mM) added to buffer A. All purification steps were followed by SDS–PAGE 12% and the final protein concentration was evaluated by measuring the UV⁄ vis absorbance at 280 nm (theoretical extinction coefficient of 1.352 M^−1 ^cm^−1^ according to ProtParam server, available in (http://web.expasy.org/protparam/). The purified protein was stored in 30% glycerol at −80 °C until use.

#### Screening of thiazolidinedione derivatives by thermal shift assay (TSA)

TSA assays were performed on Applied Biosystems 7500 RT-PCR (Applied Biosystems, Foster City, CA USA), fitted with custom filter sets. All assays were carried out in triplicate on a 96-well PCR plate (PCR plates 96 well BioRad^®^), manually sealed with transparent capping strips (Flatcap strips BioRad^®^). The plates were centrifuged for 2 min, 2000 rpm, at 25 °C and then the fluorescence of SYPRO^TM^ orange (S6650) was monitored (excitation wavelength= 492 nm and emission wavelength= 610 nm), while the plate was heated from 25 to 85 °C, in increments of 1 °C per minute.

The TSA conditions employed in this work were previously optimised^16^. Briefly, 5 µl of *Pa*PhzS (5 µM final concentration), 1 µl of SYPRO^TM^ orange (5X), 13 µl of assay buffer (50 mM potassium phosphate, 300 mM NaCl, pH 8), and 1 µl DMSO (5% v/v), as a negative control, or the putative inhibitor (diluted in DMSO) were added to each well. All experiments were carried out in triplicate

Raw fluorescence data were recorded within the Applied Biosystems 7500 Software v2.0, and then exported to NAMI^52^ for Tm calculation by the first-derivative method. Differences between Tm values (Δ*Tm*) were considered statistically significant when *p* < .01, according to the One-way ANOVA followed by Dunnett’s post-test for multiple comparisons, available in GraphPad Prism 8.0 software (GraphPadV^®^ Software, San Diego, CA, USA, www.graphpad.com).

#### Mode of inhibition by TSA

The mode of inhibition (competitive, non-competitive, uncompetitive) was evaluated by comparison to the thermal signatures described by Lea and co-workers^53^. Hence, the Δ*Tm* due to different concentrations of each thiazolidinedione derivative was employed to build sigmoid dose-response curves (APO-Tm curves), using non-linear regression (three parameters), available in GraphPad Prism 8.0 software (GraphPad^®^ Software, San Diego, CA, USA, www.graphpad.com) (Equation (1)).

$$Y=\mathrm{Bottom}+(\mathrm{Top}-\mathrm{Bottom})/(1+10^{\left(\mathrm{LogEC}50-X\right)})$$

(1)


where *Y* represents the biological response, *Bottom* stands for the minimum inhibition observed, *Top* is the maximum inhibition achieved and *X* is the concentration of the compound, on a molar scale, that affords each *Y* value.

Similarly, Δ*Tm* obtained in the presence of NADH and NAD+ (0.6 mM), and phenazine-1-carboxylic acid (PCA) (1.0 mM) were employed to calculate the NADH-Tm, NAD+-Tm and the PCA-Tm curves. All measurements were carried out in triplicate and the sigmoid curves for each experiment were compared visually.

#### Compounds’ affinity calculation by TSA

The effect of ligand’s concentration (ranging from 3.12 to 200 µM) over the *Pa*PhzS Tm (5 µM final concentration) was evaluated using the same parameters described in section (2.2.2). Next, the fraction of unfolded *Pa*PhzS at 43 °C were employed to calculate the compounds Kd, using the DSFit software (default parameters), as described by Bai and co-workers^54^. The Δ*Cp* value (0.69) required for this calculation was predicted by hydrophobic surface area-based method available on http://www.ibi.vu.nl/programs/bicepwww/. Briefly, the heat capacity (*C*) was calculated at two temperatures: 25 °C (before PhzS unfolding [*T*1]) and 50 °C (after PzhS unfolding [*T*2]) using Equation (2).

C = 332.66 ± 18.9 X (273.15 − T) + 52089 ± 1353

(2)


where *T* stands for temperature in Kelvin. Therefore *T*1 = 298.15 K, *T*2 = 323.15 K.

#### Compounds’ affinity to covalently modified *Pa*PhzS

*Pa*PhzS (2 mg/mL) was incubated with fluorescein-5-isothiocyanate (FITC) dye (6 mg/mL) containing 50 mM HEPES (pH 8.0) for 2 h, at 25 °C. Then, the solution was loaded on a Hi-Trap HP desalting column (GE Healthcare), previously equilibrated with 50 mM HEPES buffer (pH 8) and 2 CV of the same buffer were injected. The absorbance of the collected fractions was monitored at 280 nm and 490 nm and those with a molar ratio ranging from 0.3 and 1.0^55^ were employed for the Kd calculation experiments, as follows: The fluorescence of *Pa*PhzS-FITC, at 25 °C, using in a real-time thermocycler (AppliedBiosystem7500) equipped with the FAN filter (excitation 498 nm and emission 530 nm wavelengths) for 10 min in the presence of different concentrations of each compound (3.12–100.0 µM). The Raw fluorescence data, recorded within the Applied Biosystems 7500 Software v2.0, was employed for non-linear regression analysis (three parameters) (Equation 1), as available in GraphPad Prism 8.0 software (GraphPadV^®^ Software, San Diego, CA, USA, www.graphpad.com). All experiments were carried out in triplicate using 96-well PCR plate (PCR plates 96 well BioRad^®^), manually sealed with transparent capping strips (Flatcap strips BioRad^®^).

## Results

### Screening and Kd determination of thiazolidinone derivatives

**Figure 3.**
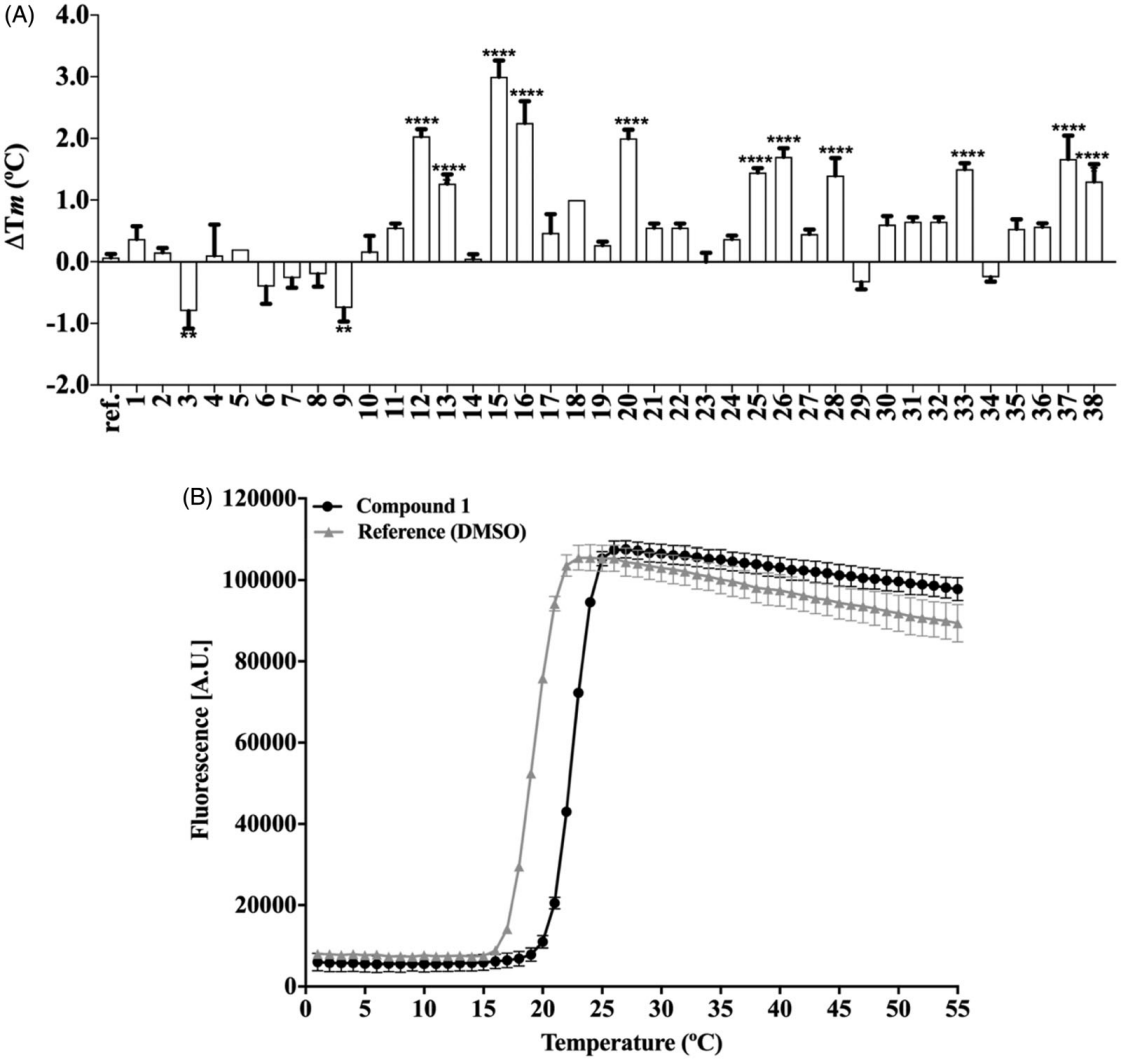
(A) Single-dose screening of thiazolidinedione derivatives (50 µM) against PhzS from *P. aeruginosa*, by TSA. The Δ*Tm* values represents the mean ± SD of the changes in Tm of each compounds (**p < .01, ****p < .0001 compared with reference (DMSO)); (B) Raw data from TSA. All data represent the mean ± SD from three independent experiments.

**Figure 4.**
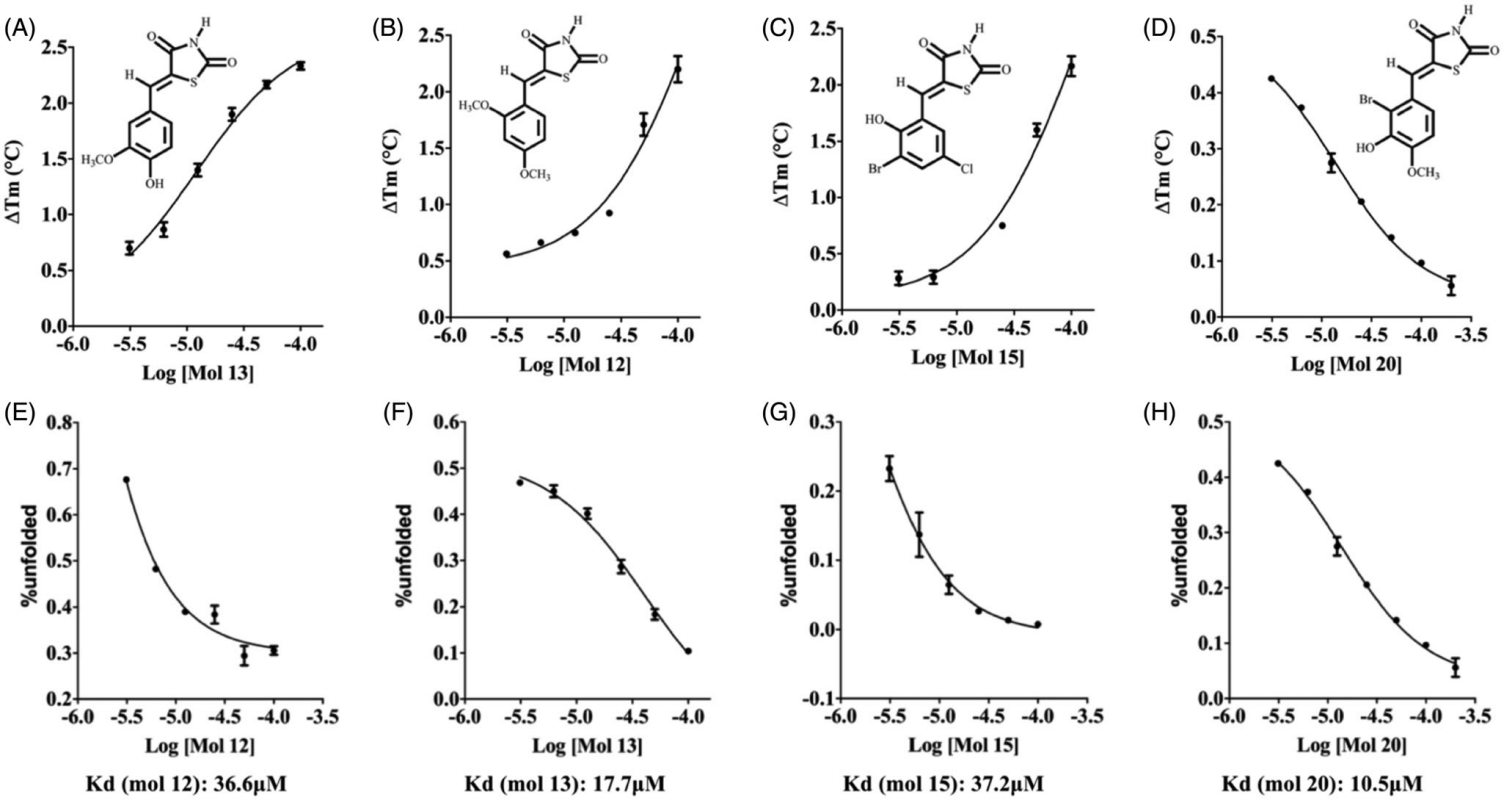
Effect of different concentrations of compounds **12**, **13**, **15** and **20** over the *Pa*PhzSTm values (upper panel – A–D) and over the fraction on unfolded *Pa*PhzS (lower panel – E–H) at 43 C. Δ*Tm* was calculated in comparison to DMSO (control) and Kd values were calculated using DSFit software. Plots were generated with GraphPad Prism v.8.0. All data represent the mean ± SD from three independent experiments.

TSA carried out at a single-concentration (50 µM) shows that approximately 60% of the compounds increase PhzS Tm value more than 0.5 °C, however, this effect is statistically significant just for compounds **3, 9 12, 13, 15, 16, 20, 25, 26, 28, 33, 37** and **38** (Figure 3). Compounds with negative ΔTm were not considered further. No compound substituted in N1 affords a significant change in *Pa*PhzS Tm. Apart from compound **33**, the replacement of benzylidene ring by fused-rings (**30–35**) affords compounds that do not bind to *Pa*PhzS. In order to reduce the number of false-positive hits identified in the initial screening, the effect of compounds **12, 13, 15, 16, 20, 25, 26, 28, 33, 37** and **38** were also evaluated at different concentrations. Among these compounds, **12, 13, 15, 16**, **20, 28 and 33** showed a clear concentration-response profile (Figure 4 and Figure 1(S) – Supplementary material), which excludes a non-specific interaction to *Pa*PhzS.

On the other hand, compounds **25** and **26** displayed a flat response in the concentration range investigated (3.12–100.0 µM) (Figure 1S – Supplementary material). Considering that **25** and **28** differ only at the substitution pattern at one position (2,3,4 OCH_3_ vs. 1,3,4 OCH_3_, respectively), it seems that substituents at *meta* play a crucial role towards *Pa*PhzS binding. Compounds **37** and **38** showed a small variation in Δ*Tm*, along with poor fit (*r*^2^ < 0.7) (Figure 1S – Supplementary material) and for that reason they were discarded.

**Figure 5.**
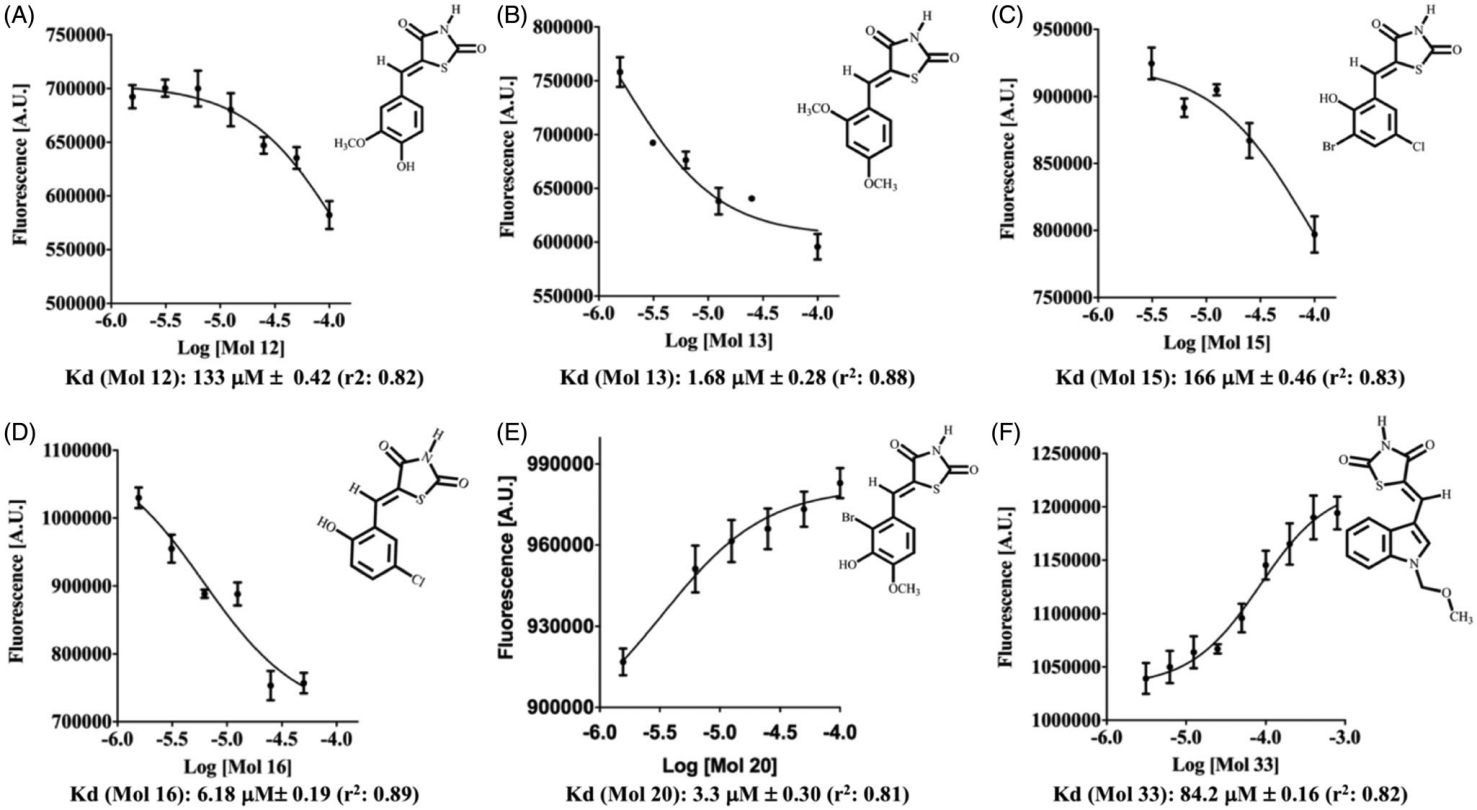
Effect of different concentrations of compounds **12, 13, 15, 16, 20** and **33** over the fluorescence signal (498 nm (excitation) and 530 nm (emission) wavelengths) of covalently labelled PhzS from *P. aeruginosa* (*Pa*PhzS-FITC). Kd values were calculated by non-linear regression as available in GraphPad Prism v.8.0. All data represent the mean ± SD from three independent experiments.

Although some authors suggest that the compounds’ affinity (Kd values) might be calculated directly from those curves with reasonable accuracy^56^, there is some concern about the thermodynamic basis for this approach^57^. Bai and co-workers devised an elegant workaround that limitation, which relies on the percentage of unfolded protein at a fixed temperature^54^. Using this approach, the Kd values were calculated at 43 °C, which is close to the *Pa*PhzS Tm value in the absence of ligands (42 °C), so that the fraction of folded and unfolded protein was close to 1,0. According to Bai and co-workers (2019) Kd values close to the protein concentration (5 µM) or lower should be taken with scepticism. However, only one compound had Kd value in this range (compound **16** -Kd = 4.2 µM). The other compounds display affinity ranging from 10.5 − 50.5 µM (compound **20** and compound **33** respectively) (Figure 4 and Supplementary material Figure 3S). As these Kd values were obtained at a temperature higher than the physiologic one, we decided to employ an orthogonal assay to evaluate the compounds’ affinity. In order to achieve this goal, *Pa*PhzS was labelled with FITC and the effects of different concentration of each inhibitor on the covalently modified *Pa*PhzS was monitored at 25 °C (Figure 5).

According to this assay, three compounds (**13, 16** and **20)** are low micromolar ligands of *Pa*PhzS, this result is in good agreement with previous results that also suggest these are the most promising compounds. The lower values of Kd obtained here might be a consequence of the reduced temperature employed in this assay. Although this assay also agrees that **12**, **15** and **33** have low affinity to *Pa*PhzS their affinity for *Pa*PhzS-FITC is even lower than calculated by DSFit for *Pa*PhzS. Since **13** and **28** differ by one OCH3, it is tentative to assume that this moiety causes steric hindrance and abolishes the activity. In fact, no compound is substituted, simultaneously, at R3 and R4 is active. Moreover, comparison of compound **15** and **16** Kd values (166 µM ± 0.46 vs. 6.18 µM ± 0.19, respectively) and compound **19** (Δ*Tm* close to zero) supports the hypothesis that di-substitution at meta position is detrimental to the affinity.

### Inhibition mode determination by TSA

**Figure 6.**
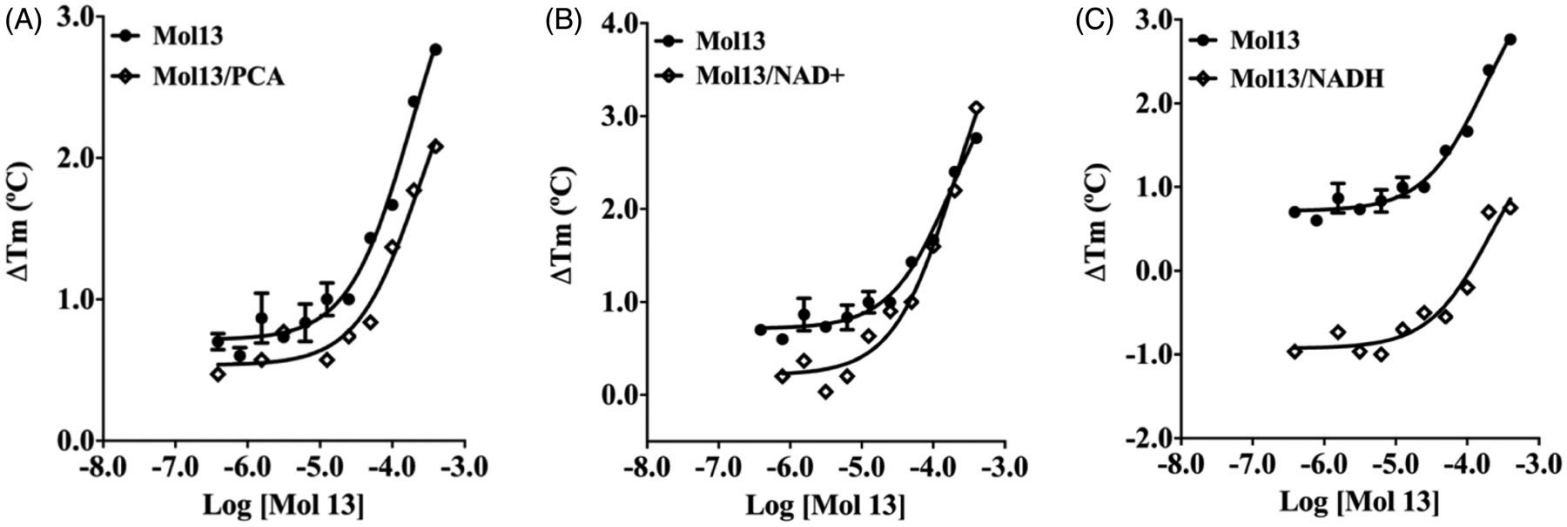
Thermal shift signatures of compound 13 in presence/absence of PCA (A) or NADH (B) or NAD+ (C). All data represent the mean ± SD from three independent experiments.

Since the catalytic efficiency of *Pa*PhzS to convert PCA to hydroxy-phenazine is low and requires an excess of NADH (cofactor)^29^, using kinetic assays to evaluate the mode of inhibition might be misleading. Considering that TSA has been employed as a suitable alternative to achieve this goal^53^^,^^58–60^, we employed this approach to confirm that the most promising compound (**13**) binds to the cofactor binding site, through the analysis of its thermal shift signature in the presence and absence of either the cofactor to the substrate (Figure 6).

Previously, we have shown that both the substrate and the cofactor cause a concentration-dependent decrease in *Pa*PhzS’s Tm value^11^. Then, the ΔTm curves of *Pa*PhzS in the presence of a fixed concentration of either NADH (0.6 mM) or PCA (1.0 mM are displaced south of the curve in their absence. Nevertheless, in the presence of either NADH or PCA, the curves are parallel to those seen in their absence. This profile is the opposite of the one described by Lea and co-workers,^53^ but the overall analysis of the thermal signature holds: The parallel curves observed for compound **13** in the presence and absence of the PCA/NADH are compatible with the non-competitive mode of inhibition towards both the substrate and cofactor. This result might seem unexpected, once thiazolidinedione derivatives were expected to mimic the binding profile of the purine ring from NADH. One reasonable explanation is that FAD is readily reduced to FADH in the presence of NADH (Data not shown), and then NAD + leaves the binding site, thus not affecting compound **13’s** affinity to *Pa*PhzS. In order to support this hypothesis, we avoided the conformational shift that would expel NAD + from its binding site, by performing the assay in the presence of NAD+. As anticipated, the curves get closer as the concentration of compound **13** increases, suggesting a competitive mode of inhibition to this compound and corroborating its binding profile to the cofactor binding site.

## Discussion

The development of novel antibiotic drugs continues to be of utmost importance^61^^,^^62^. However, the research and development of novel antibiotic drugs by the Big-Pharma industries has steadily declined in the last decades^63^^,^^64^. There are some promising alternatives to deal with this issue^65^^,^^66^. Among them, anti-virulence drug development relies on the hypothesis that is better to “live in peace with the enemy” than to kill it^67^. One way to achieve this goal is by rendering the pathogenic bacteria harmless to the human host and assuming this process will put less evolutive pressure on the microorganism than the currently available drugs^67–70^. Much effort has been put into the inhibition of pigmented virulence factors such as staphyloxanthin and pyocyanin^71^^,^^72^ but the approach is taken to their modulation is rather distinct. The first relies on the inhibition of druggable targets that are responsible for staphyloxanthin biosynthesis,^73^^,^^74^ whereas the second is focussed on the modulation of quorum sensing mechanisms that lie upstream the biosynthetic pathway that builds up this virulence factor^75–78^.

According to Ni and co-workers (2019),^71^ targeting enzymes from the pyocyanin biosynthesis pathway is a valid and underexplored strategy. In accordance with this standpoint of view, it has been proposed that *Pa*PhzS is a druggable target^28^ and we have shown that thiazolidine-2,4-dione derivatives, bearing a dihydroquinazoline ring at the *para* position of the benzylidene ring, have a micromolar affinity to PhzS from *P. aeruginosa* and reduce pyocyanin production without a significant impact on the cell growth rate^11^. The most promising compound (Figure 1(A)) might be employed for hit-to-lead optimisation efforts, but a careful analysis of its ligand efficiency argues otherwise. Molecular obesity has been one of the largest problems in drug development campaigns in the last decades^79^^,^^80^ and our initial effort to identify *Pa*PhzS inhibitors seems to suffer from the same problem. In general, hits with LE> 0.30 have a higher success rate than those with lower LE, since this metric is highly correlated to high steric and electronic complementarity within the binding site. The low LE value for the previous hit (**1A**- LE= 0.21) suggests that some moieties are detrimental for it to fit into the binding site. This comes as no surprise, once molecular complexity is inversely related to LE^81^. Hence, an obvious alternative to increase LE is to strip the molecules from auxophoric groups and to focus on the chemical features that are essential for biological activity. Considering that previous work suggests the thiazolidine-2,4-dione ring mimics the cofactor binding profile, it was decided to evaluate whether N1- substituted compounds would bind to *Pa*PhzS. The lack of activity seen in N1-substituted compounds (**1–7**) supports that the H-bonding capability of the nitrogen within the thiazolidine-2,4-dione ring is essential for *Pa*PhzS binding. This result is in good agreement with the morphological similarity analysis carried out for dihydroquinazoline substituted thiazolidine-2,4-dione derivatives^11^.

Next, the replacement of the central phenyl by fused heteroaromatic rings was probed. In contrast to N1 substitution, this strategy affords compounds that bind to *Pa*PhzS with two-digit micromolar affinity (i.e. **33** *Pa*PhzS-FITC-Kd = 84.2 µM and DSFit-Kd > 50). However, additional studies are required to settle once and for all if this is a promising lead-optimisation strategy since the substitution pattern on this ring remains unexplored. Following a more conservative approach, it was decided to replace dihydroquinazoline moiety with less-bulky substituents. This strategy afforded compounds with lower affinity, but with higher LE (**12** and **15**) than the initial hit (Figure 1(A)) (Table 3).

**Table 3.** The binding affinity (Kd), ligand efficiency (LE)* and binding efficiency index (BEI)^#^ of *Pa*PhzS inhibitors.

DSFit software				FITC		
	Kd (μM)	LE	BEI	Kd (μM)	LE	BEI
1A (previous hit)	9.3	0.21	11.0	18.0**	0.21	10.4
Mol 12	36.6	0.36	17.6	133 ± 0.4	0.32	15.4
Mol 13	17.7	0.37	17.9	1.68 ± 0.2	0.44	21.8
Mol 15	37.2	0.36	13.3	166 ± 0.5	0.31	11.4
Mol 16	4.2	0.47	21.0	6.18 ± 0.2	0.45	20.4
Mol 20	10.5	0.38	15.0	3.3 ± 0.3	0.42	16.6
Mol 33	50.0	0.30	14.9	84.2 ± 0.2	0.28	14.1

*LE: 1.4*pKd/HA, where heavy (non-hydrogen) atoms [82].

^#^BEI: (pKd/MW)×1000, where MW is molecular weight (Da) [83].

**Kd value calculated by microscale thermophoresis [11].

One might argue that LE provides an unfair advantage for fragment-like compounds such as those reported here, especially for compounds bearing halogen substituents. This concern is, at least partially, taken into account by the binding efficiency index^83^, which considers the compounds’ molecular weight instead of their heavy-atom count. When this metric is employed compound **15** displays the lowest BEI among the thiazolidine-2,4-dione derivatives reported in this work, but its value remains in the same range as the previous hit (BEI **1A** = 10.4 vs. **15 **=** **11.4). On the other hand, both assays show a significant improvement in the affinity and efficiency of **13** (Kd 1.68, LE = 0.45, BEI 21.79), **16** (Kd 6.18 LE = 0.46, BEI 20.42) and **20** (kd LE = 0.43, BEI 16.71). In an attempt to relate the structural changes in compounds with their putative interactions within the binding pocket, the difference in free energy of binding among those compounds (ΔΔ*G*_binding_ = RTln(Kd^a^/Kd^b^) where Kd^a^ and Kd^b^ represent the affinity of two compounds being compared, *R* = 8.31 J/Kmol and *T* = 298.15 K) can be calculated. ΔΔ*G*_binding_ between compounds **12** and **13**, using *Pa*PhzS-FITC-Kd values, yields a difference that is compatible with an H-bond (2.6 Kcal/mol). Similar values are seen for the comparison between compounds **13** and **15** (2.7 Kcal/mol), compounds **12** and **20** (2.2 Kcal/mol) and compounds **15** and **20** (2.3 Kcal/mol). These values support the importance of H-bonding ability to potency. This feature might be correlated with the keto-enol tautomerism as well as the planarity between the 5-arylidene and the thiazolidine-2,4-dione moieties, which are affected by electron-donating/accepting substituents decorating the ring^84^. In fact, *para* electron-accepting groups that stabilise the keto-form only afford inactive compounds (**8–11**), and except for compound **20**, the same can be stated for substituents in the *ortho* position (either R1 or R5). On the other hand, steric restraints also play a key role for thiazolidine-2,4-dione binding affinity, as observed for *meta* disubstituted compounds: **19** and **25** are inactive, whereas **15** shows reduced affinity in comparison to **13**, **16** or **20**, all of which have only one substitution at *meta* position.

When molecular simplification is carried out, conformational flexibility may cause the novel compounds to adopt new bioactive conformations or even explore small binding clefts that were inaccessible at first. The results provided by the mode of inhibition studies suggest this is not the case for the compounds reported in this work and suggests the same structure-activity relationships hold for the whole series. Last but not least, careful analysis of TSA raw curves (Figure 2S – Supplementary material) hint that these compounds do not behave as classical PAINS^85^, which reinforces their usefulness as a promising scaffold to design novel mechanism-based inhibitors of *Pa*PhzS.

## Conclusion

One approach to overcome the dilemma relies on bacterial virulence modulation. Although pyocyanin is a virulence factor from *Pseudomonas aeruginosa* employed as an end-point in most anti-virulence drug development projects, the enzymes responsible for its biosynthesis remain largely unexplored as potential therapeutic targets. The results described here show that unsubstituted N1 at the thiazolidine-2,4-dione ring is essential for *Pa*PhzS inhibition and that molecular simplification of (E)-5–(4-((4-oxo-3-phenyl-3,4-dihydroquinazolin-2-yl)methoxy)benzylidene)thiazolidine-2,4-dione affords improved lead compounds that retain the original mode of inhibition (competitive to NAD+). Moreover, steric clashes due to simultaneous substitution at both meta positions are detrimental to 5-arylidene-thiazolidine-2,4-dione derivatives affinity. However, there seems to be some plasticity within *Pa*PhzS binding site, since the replacement of benzyl ring by indole substituted ring does not abolish the affinity. Taken together, this information paves the way to develop the third round of *Pa*PhzS inhibitors with improved potency.

## Supplementary Material

Supplemental Material

## References

[1] Tomašić T, Peterlin Mašič L. Rhodanine as a scaffold in drug discovery: a critical review of its biological activities and mechanisms of target modulation. Expert Opin Drug Discov 2012;7:549–60. 22607309 10.1517/17460441.2012.688743

[2] Tripathi AC, Gupta SJ, Fatima GN, et al. 4-Thiazolidinones: the advances continue…. Eur J Med Chem 2014;72:52–77. 24355348 10.1016/j.ejmech.2013.11.017

[3] Mendgen T, Steuer C, Klein CD. Privileged scaffolds or promiscuous binders: a comparative study on rhodanines and related heterocycles in medicinal chemistry. J Med Chem 2012;55:743–53. 22077389 10.1021/jm201243p

[4] Welsch ME, Snyder SA, Stockwell BR. Privileged scaffolds for library design and drug discovery. Curr Opin Chem Biol 2010;14:347–61. 20303320 10.1016/j.cbpa.2010.02.018 PMC2908274

[5] Willson TM, Cobb JE, Cowan DJ, et al. The structure-activity relationship between peroxisome proliferator-activated receptor gamma agonism and the antihyperglycemic activity of thiazolidinediones . J Med Chem 1996;39:665–8. 8576907 10.1021/jm950395a

[6] Ramirez MA, Borja NL. Epalrestat: an aldose reductase inhibitor for the treatment of diabetic neuropathy. Pharmacotherapy 2008;11:231–5. 10.1592/phco.28.5.646 18447661

[7] Leite FHA, Santiago PD, Froes TQ, et al. Structure-guided discovery of thiazolidine-2,4-dione derivatives as a novel class of Leishmania major pteridine reductase 1 inhibitors . Eur J Med Chem 2016;123:639–48. 27517809 10.1016/j.ejmech.2016.07.060

[8] Neri FSM, Costa JD, Froes TQ, et al. Antileishmanial activity evaluation of thiazolidine-2,4-dione against *Leishmania infantum* and *Leishmania braziliensis*. Parasitol Res 2020;119:2263–74. 32462293 10.1007/s00436-020-06706-3

[9] Bhattarai BR, Kafle B, Hwang JS, et al. Thiazolidinedione derivatives as PTP1B inhibitors with antihyperglycemic and antiobesity effects. Bioorganic Med Chem. Lett 2009;19:6161–5. 10.1016/j.bmcl.2009.09.020 19783142

[10] Bhat BA, Ponnala S, Sahu DP, et al. Synthesis and antihyperglycemic activity profiles of novel thiazolidinedione derivatives. Bioorg Med Chem 2004;12:5857–64. 15498661 10.1016/j.bmc.2004.08.031

[11] Froes TQ, Guido RVC, Metwally K, Castilho MS. A novel scaffold to fight *P. aeruginosa* pyocyanin production: early-steps to novel anti-virulence drugs. Future Med Chem 2020;16:104155. 10.4155/fmc-2019-0351 32772556

[12] Access to Medicine Foundation. Antimicrobial Resistance Benchmark 2018. (2018). Available from: https://accesstomedicinefoundation.org/media/uploads/downloads/5c46f0d1cbefe_Antimicrobial-Resistance-Benchmark-2018.pdf

[13] Centers for Disease Control and Prevention. Antibiotic Use in the United States. 2017: Progress and Opportunities. Available from: https://www.cdc.gov/antibiotic-use/stewardship-report/pdf/stewardship-report.pdf

[14] O’Neill J. Securing new drugs for future generations: the pipeline of antibiotics – review on antimicrobial resistance. Rev Antimicrob Resist 2016;44:1–44.

[15] Ventola CL. The antibiotic resistance crisis: part 1: causes and threats. P T A Peer-Reviewed J Formul Manag 2015;40:277–83. PMC4378521 25859123

[16] World Health Organization. Global action plan on antimicrobial resistance. (2015). Available from: https://apps.who.int/iris/bitstream/handle/10665/193736/9789241509763_eng.pdf?sequence=1 10.7196/samj.9644 26242647

[17] Ali J, Rafiq QA, Ratcliffe E. Antimicrobial resistance mechanisms and potential synthetic treatments. Futur Sci OA 2018;4:FSO290. 10.4155/fsoa-2017-0109 PMC5905577 29682325

[18] Clatworthy AE, Pierson E, Hung DT. Targeting virulence: a new paradigm for antimicrobial therapy. Nat Chem Biol 2007;3:541–8. 17710100 10.1038/nchembio.2007.24

[19] Rasko DA, Sperandio V. Anti-virulence strategies to combat bacteria-mediated disease. Nat Rev Drug Discov 2010;9:117–28. 20081869 10.1038/nrd3013

[20] Shoham M, Greenberg M. Preventing the spread of infectious diseases: antivirulents versus antibiotics. Future Microbiol 2017;12:365–8. 28339290 10.2217/fmb-2017-0011

[21] Forezi LSM, Froes TQ, Cardoso MFC, et al. Synthesis and biological evaluation of coumarins derivatives as potential inhibitors of the production of *Pseudomonas aeruginosa* virulence factor pyocyanin. Curr Top Med Chem 2018;18: 149–56. 29595112 10.2174/1568026618666180329122704

[22] Müh U, Schuster M, Heim R, et al. Novel *Pseudomonas aeruginosa* quorum-sensing inhibitors identified in an ultra-high-throughput screen. Antimicrob Agents Chemother 2006;50:3674–9. 16966394 10.1128/AAC.00665-06 PMC1635174

[23] Paczkowski JE, Mukherjee S, McCready AR, et al. Flavonoids suppress *Pseudomonas aeruginosa* virulence through allosteric inhibition of quorum-sensing receptors. J Biol Chem 2017;292:4064–76. 28119451 10.1074/jbc.M116.770552 PMC5354481

[24] Defoirdt T. Quorum-sensing systems as targets for antivirulence therapy. Trends Microbiol 2018;26:313–28. 29132819 10.1016/j.tim.2017.10.005

[25] Fong J, Mortensen KT, Nørskov A, et al. Itaconimides as novel quorum sensing inhibitors of *Pseudomonas aeruginosa*. Front Cell Infect Microbiol 2018;8:443–11. 30666301 10.3389/fcimb.2018.00443 PMC6330316

[26] European Centre for Disease Prevention and Control. Surveillance of antimicrobial resistance in Europe Annual report of the European Antimicrobial Resistance Surveillance Network (EARS-Net) 2017.

[27] Lau GW, Ran H, Kong F, et al. *Pseudomonas aeruginosa* pyocyanin is critical for lung infection in mice. Infect Immun 2004;72:4275–8. 15213173 10.1128/IAI.72.7.4275-4278.2004 PMC427412

[28] Froes TQ, Baldini RL, Vajda S, Castilho MS. Structure-based druggability assessment of anti-virulence targets from *Pseudomonas aeruginosa*. Curr Protein Pept Sci 2019;20:1189–203. 31038064 10.2174/1389203720666190417120758 PMC7067568

[29] Greenhagen BT, Shi K, Robinson H, et al. Crystal structure of the pyocyanin biosynthetic protein PhzS. Biochemistry 2008;47:5281–9. 18416536 10.1021/bi702480t

[30] Blankenfeldt W, Parsons JF. The structural biology of phenazine biosynthesis. Curr Opin Struct Biol 2014;29:26–33. 25215885 10.1016/j.sbi.2014.08.013 PMC4268259

[31] Romagnoli R, Baraldi PG, Salvador MK, et al. Anticancer activity of novel hybrid molecules containing 5-benzylidene thiazolidine-2,4-dione. Eur J Med Chem 2013;63:544–57. 23537942 10.1016/j.ejmech.2013.02.030

[32] Barros CD, Amato AA, Oliveira T. d, et al. Synthesis and anti-inflammatory activity of new arylidene-thiazolidine-2,4-diones as PPARgamma ligands. Bioorg Med Chem 2010;18:3805–11. 20471839 10.1016/j.bmc.2010.04.045

[33] Ates-Alagoz Z, Altanlar N, Buyukbingol E. Synthesis of 4-substituted 2- (4-Methylpiperazino) pyrimidines and quinazoline analogs as serotonin 5-HT 2A receptor ligands. J Heterocycl Chem 2009;46:1259–65.

[34] Tuncbilek M, Altanlar N. Synthesis of new 3-(substituted phenacyl)-5-[3'-(4H-4-oxo-1-benzopyran-2-yl)-benzylidene]-2,4-thiazolidinediones and their antimicrobial activity . Arch Pharm 2006;339:213–6. 10.1002/ardp.200500180 16572478

[35] Alhameed RA, Almarhoon Z, Bukhari SI, et al. Synthesis and antimicrobial activity of a new series of thiazolidine-2,4-diones carboxamide and amino acid derivatives. Molecules 2019;25:105–17. 10.3390/molecules25010105 PMC6983134 31892119

[36] Tahlan S, Verma PK. Synthesis, SAR and *in vitro* therapeutic potentials of thiazolidine-2,4-diones. Chem Cent J 2018;12:1–11. 30515635 10.1186/s13065-018-0496-0 PMC6768028

[37] Liu Y, Li R, Xing Y. A simple, efficient and green procedure for knoevenagel condensation in hydroxy-functionalized ionic liquids. Heterocycles 2015;91:1385–97.

[38] De Paiva RKC, Da Silva JF, Moreira HA, et al. Synthesis, antimicrobial activity and structure-activity relationship of some 5-arylidene-thiazolidine-2,4-dione derivatives. J Braz Chem Soc 2018;30:164–72.

[39] Khan FAK, Patil RH, Shinde DB, Sangshetti JN. Design and synthesis of 4'-((5-benzylidene-2,4-dioxothiazolidin-3-yl)methyl)biphenyl-2-carbonitrile analogs as bacterial peptide deformylase inhibitors. Chem Biol Drug Des 2016;88:938–44. 27401234 10.1111/cbdd.12817

[40] Azad L, Ghazvini M, Sanaeishoar H, Yavari I. Synthesis of functionalized 1,2-dihydroisoquinolines via one-pot reactions of isoquinoline, alkyl propiolate, and thiazolidin-2,4-dione. J Chem Res 2019;43:457–60.

[41] Da Silva IM, Da Silva Filho J, Santiago P, et al. Synthesis and antimicrobial activities of 5-arylidene-thiazolidine-2,4- dione derivatives. Biomed Res Int 2014;2014:316082. 24895565 10.1155/2014/316082 PMC4033545

[42] Zhang Y, Zhou Z. A solvent-free protocol for the green synthesis of 5-arylidene-2,4-thiazolidinediones using ethylenediamine diacetate as catalyst. Org Chem Int 2012;2012:1–5.

[43] Sharma H, Lather V, Grewal AS, Pandita D. Synthesis, anti-inflammatory activity and docking studies of some newer 1,3-thiazolidine-2,4-dione derivatives as dual inhibitors of PDE4 and PDE7. Curr Comput Aided Drug Des 2019;15:225–34. 30280674 10.2174/1573409914666181003151528

[44] Mohanty S, Roy AK, Sandeep Reddy G, et al. Knoevenagel condensation of aromatic bisulfite adducts with 2,4-thiazolidinedione in the presence of Lewis acid catalysts. Tetrahedron Lett 2015;56:2564–7.

[45] Palkar M, Jalalpure S, Rane R, et al. novel series of coumarinyl substituted-thiazolidin-2,4-dione analogs as anticancer agents: design, synthesis, spectral studies and cytotoxicity evaluation. Anticancer Agents Med Chem 2015;15:970–9. 25909852 10.2174/1871520615666150424110339

[46] Kaarsholm B, Christian N, Olsen B. Preparation of novel ligands for the HisB10 Zn2+ sites of the R-state insulin hexamer and their use in pharmaceutical preparations comprising insulin. PCT Int. Appl. 2006;3–7:WO 2006082245 A1 20060810.

[47] Chandrappa S, Vinaya K, Prasanna DS, Rangappa KS. Mild and highly efficient method for the synthesis of arylidenethiazolidinone analogues. Proc Indian Natl Sci Acad 2011;77:343–9.

[48] Sato S, Shirakawa S, Tatsui A, et al. Thiazolidine derivatives as chymase inhibitors and prophylactic and therapeutic drugs containing them for cardiovascular diseases. Jpn Kokai Tokkyo Koho 2000; JP 2000095770 A 20000404.

[49] Maccari R, Ottanà R, Curinga C, et al. Structure-activity relationships and molecular modelling of 5-arylidene-2,4-thiazolidinediones active as aldose reductase inhibitors. Bioorg Med Chem 2005;13:2809–23. 15781392 10.1016/j.bmc.2005.02.026

[50] Libermann D, Himbert J. HL. La thiazolidione, point de depart d’une synthèse des acides thiopyruviques et thioglyoxiliques substituèes. Bull Soc Chim Fr 1948;11–12:1120–4.

[51] Albuquerque JF, Azevedo LC, Galdino SL, Chantegrel J, et al. Synthesis and structural study of 5-arylidene thiazolidine-2,4-diones and 3-substituted-4-thio-imidazolidine-2-ones. Ann Pharm Fr 1995;53:209–14. 7503509

[52] Grøftehauge MK, Hajizadeh NR, Swann MJ, Pohl E. Protein-ligand interactions investigated by thermal shift assays (TSA) and dual polarization interferometry (DPI). Acta Crystallogr D Biol Crystallogr 2015;71:36–44. 25615858 10.1107/S1399004714016617 PMC4304684

[53] Lea WA, Simeonov A. Differential scanning fluorometry signatures as indicators of enzyme inhibitor mode of action: case study of glutathione s-transferase. PLOS One 2012;7:e36219. 22558390 10.1371/journal.pone.0036219 PMC3340335

[54] Bai N, Roder H, Dickson A, Karanicolas J. Isothermal analysis of thermofluor data can readily provide quantitative binding affinities. Sci Rep 2019;9:2650–15. 30804351 10.1038/s41598-018-37072-x PMC6389909

[55] The TH, Feltkamp TEW. Conjugation of fluorescein isothiocyanate to antibodies I. Experiments on the conditions of conjugation. Immunology 1970;18:865–73. 5310665 PMC1455721

[56] Vivoli M, Novak HR, Littlechild JA, Harmer NJ. Determination of protein-ligand interactions using differential scanning fluorimetry. J Vis Exp 2014;91:1–13. 10.3791/51809 PMC4692391 25285605

[57] Cimmperman P, Baranauskiene L, Jachimoviciūte S, et al. A quantitative model of thermal stabilization and destabilization of proteins by ligands. Biophys J 2008;95:3222–31. 18599640 10.1529/biophysj.108.134973 PMC2547457

[58] Niesen FH, Schultz L, Jadhav A, et al. High-affinity inhibitors of human NAD+-dependent 15-hydroxyprostaglandin dehydrogenase: mechanisms of inhibition and structure-activity relationships. PLoS One 2010;5:e13719. 21072165 10.1371/journal.pone.0013719 PMC2970562

[59] Auld DS, Lovell S, Thorne N, et al. Molecular basis for the high-affinity binding and stabilization of firefly luciferase by PTC124. Proc Natl Acad Sci U S A 2010;107:4878–83. 20194791 10.1073/pnas.0909141107 PMC2841876

[60] Simeonov A. Recent developments in the use of differential scanning fluorometry in protein and small molecule discovery and characterization. Expert Opin Drug Discov 2013;8:1–20. 23738712 10.1517/17460441.2013.806479 PMC4906806

[61] World Health Organization. . Antibacterial agents in clinical development: an analysis of the antibacterial clinical development pipeline. 2019. Available from: https://apps.who.int/iris/bitstream/handle/10665/330420/9789240000193-eng.pdf

[62] Renwick MJ, Brogan DM, Mossialos E. A systematic review and critical assessment of incentive strategies for discovery and development of novel antibiotics. J Antibiot 2016;69:73–88. 10.1038/ja.2015.98 PMC4775540 26464014

[63] Renwick M, Mossialos E. What are the economic barriers of antibiotic R&D and how can we overcome them? Expert Opin Drug Discov 2018;13:889–92. 30175625 10.1080/17460441.2018.1515908

[64] Dheman N, Mahoney N, Cox EM, et al. An analysis of antibacterial drug development trends in the United States, 1980–2019. Clin Infect Dis 2020;72:1–7. 10.1093/cid/ciaa859 32584952

[65] Hoffman PS. Antibacterial discovery: 21st century challenges. Antibiotics 2020;9:1–10. 10.3390/antibiotics9050213 PMC7277910 32353943

[66] Calvert MB, Jumde VR, Titz A. Pathoblockers or antivirulence drugs as a new option for the treatment of bacterial infections. Beilstein J Org Chem 2018;14:2607–17. 30410623 10.3762/bjoc.14.239 PMC6204809

[67] DIckey SW, Cheung GYC, Otto M. Different drugs for bad bugs: antivirulence strategies in the age of antibiotic resistance. Nat Rev Drug Discov 2017;16:457–71. 28337021 10.1038/nrd.2017.23 PMC11849574

[68] Inglis RF, Brown SP, Buckling A. Spite versus cheats: competition among social strategies shapes virulence. Pseudomonas Aeruginosa. Evolution 2012;66:3472–84. 23106711 10.1111/j.1558-5646.2012.01706.x PMC3795443

[69] El-halfawy OM, Czarny TL, Flannagan RS, et al. Discovery of an antivirulence compound that reverses β-lactam resistance in MRSA . Nat Chem Biol 2020;16:143–9. 31768032 10.1038/s41589-019-0401-8

[70] Lindsay RJ, Kershaw MJ, Pawlowska BJ, et al. Harbouring public good mutants within a pathogen population can increase both fitness and virulence. Elife 2016;5:1–25. 10.7554/eLife.18678 PMC5193496 28029337

[71] Ni S, Li B, Xu Y, et al. Targeting virulence factors as an antimicrobial approach: pigment inhibitors. Med Res Rev 2020;40:293–338. 31267561 10.1002/med.21621

[72] Liu GY, Nizet V. Color me bad: microbial pigments as virulence factors. Trends Microbiol 2009;17:406–13. 19726196 10.1016/j.tim.2009.06.006 PMC2743764

[73] Song Y, Liu CI, Lin FY, et al. Inhibition of staphyloxanthin virulence factor biosynthesis in *Staphylococcus aureus*: *in vitro*, *in vivo*, and crystallographic results. J Med Chem 2009;52:3869–80. 19456099 10.1021/jm9001764 PMC2753857

[74] Wang Y, Chen F, Di H, et al. Discovery of potent benzofuran-derived diapophytoene desaturase (CrtN) inhibitors with enhanced oral bioavailability for the treatment of methicillin-resistant *Staphylococcus aureus* (MRSA) infections. J Med Chem 2016;59:3215–30. 26999509 10.1021/acs.jmedchem.5b01984

[75] Miller LC, O’Loughlin CT, Zhang Z, et al. Development of potent inhibitors of pyocyanin production in *Pseudomonas aeruginosa*. J Med Chem 2015;58:1298–306. 25597392 10.1021/jm5015082 PMC4332565

[76] Wang M, Zhao L, Wu H, et al. Cladodionen is a potential quorum sensing inhibitor. Mar Drugs 2020;18:205–14. 10.3390/md18040205 PMC7230538 32290259

[77] Malešević M, Di LF, Filipić B, et al. *Pseudomonas aeruginosa* quorum sensing inhibition by clinical isolate Delftia tsuruhatensis 11304: involvement of N-octadecanoylhomoserine lactones. Sci Rep 2019;9:1–13. 31712724 10.1038/s41598-019-52955-3 PMC6848482

[78] Kitao T, Lepine F, Babloudi S, et al. Crossm molecular insights into function and competitive inhibition of *Pseudomonas aeruginosa* multiple virulence factor regulator. MBio 2018;9:1–13. 10.1128/mBio.02158-17 PMC5770554 29339431

[79] Polanski J, Bogocz J, Tkocz A. The analysis of the market success of FDA approvals by probing top 100 bestselling drugs. J Comput Aided Mol Des 2016;30:381–9. 27125384 10.1007/s10822-016-9912-5

[80] Elsebaei MM, Abutaleb NS, Mahgoub AA, et al. Phenylthiazoles with nitrogenous side chain: an approach to overcome molecular obesity. Eur J Med Chem 2019;182:111593. 31446245 10.1016/j.ejmech.2019.111593

[81] Hann MM, Leach AR, Harper G. Molecular complexity and its impact on the probability of finding leads for drug discovery. J Chem Inf Comput Sci 2001;41:856–64. 11410068 10.1021/ci000403i

[82] Hopkins AL, Groom CR, Alex A. Ligand efficiency: a useful metric for lead selection. Drug Discov Today 2004;9:430–1. 15109945 10.1016/S1359-6446(04)03069-7

[83] Abad-Zapatero C, Metz JT. Ligand efficiency indices as guideposts for drug discovery. Drug Discov Today 2005;10:464–9. 15809192 10.1016/S1359-6446(05)03386-6

[84] Tri N, Marinkovi A, Ran M. Spectrochimica acta part A: molecular and biomolecular spectroscopy substituent and solvent effects on intramolecular charge transfer of. Spectrochim Acta Part A 2012;86:500–7. 10.1016/j.saa.2011.10.074 22153743

[85] Redhead M, Satchell R, Swift D, et al. A combinatorial biophysical approach; FTSA and SPR for identifying small molecule ligands and PAINs. Anal Biochem 2015;479:63–73. 25837771 10.1016/j.ab.2015.03.013

