# Supplementary material for: Synthesis and biological evaluation of thiazolidinedione derivatives with high ligand efficiency to P. aeruginosa PhzS
Source: J Enzyme Inhib Med Chem. 2021 Jun 3;36(1):1217–29. doi: 10.1080/14756366.2021.1931165 (PMC8186431; doi:10.1080/14756366.2021.1931165)
Supplement: Supplemental Material [file IENZ_A_1931165_SM8475.pdf]

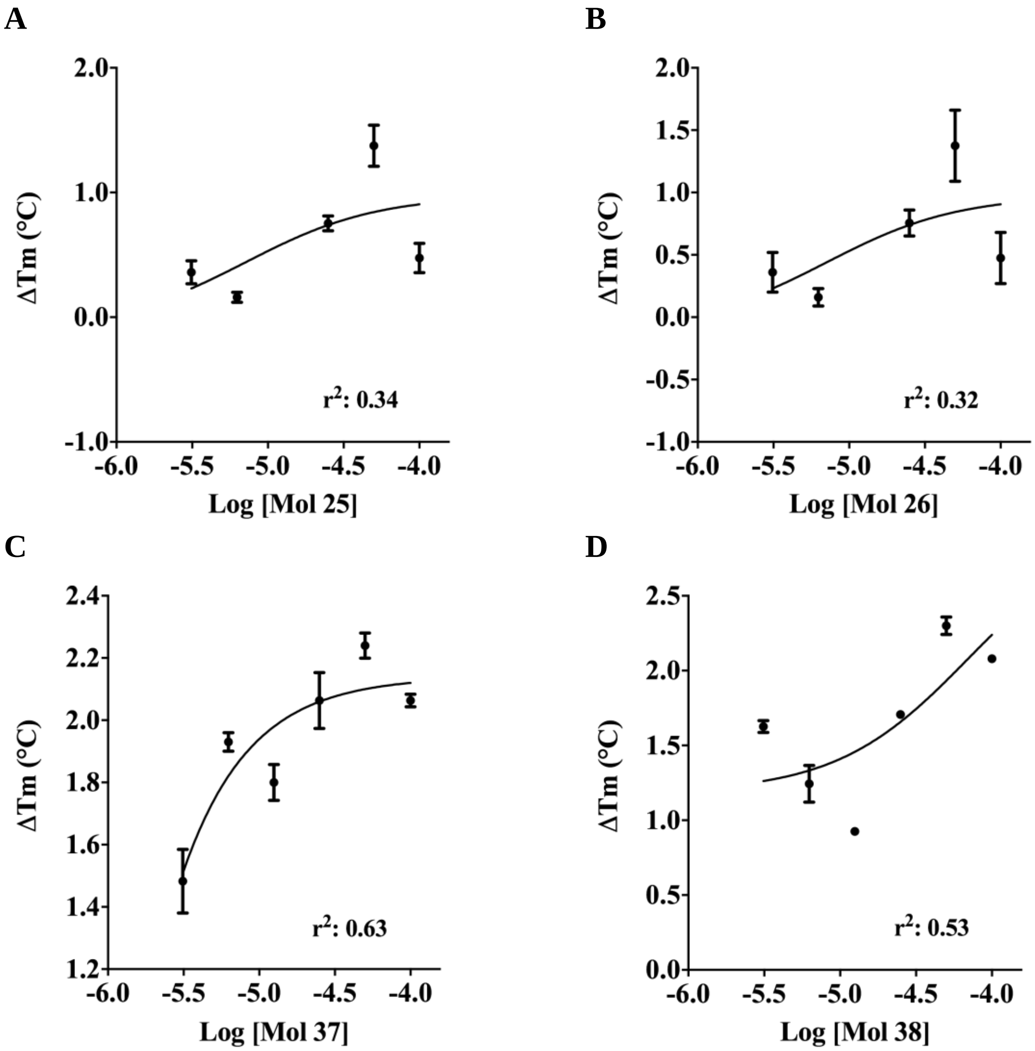

Figure 1S. Effect of different concentrations of compounds **25**, **26**, **37** and **38** over the *Pa*PhzS Tm values. Graphs were generated with GraphPad Prism v.8.0.

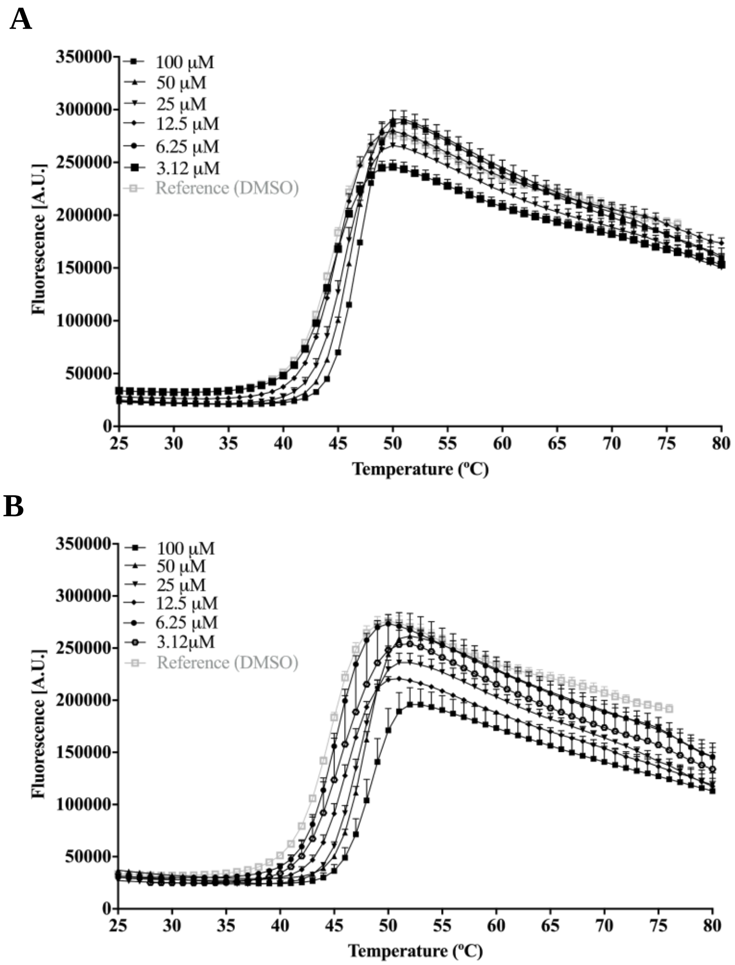

Figure 2S. Raw data from concentration-response curves obtained from TSA (A) Compound 13 (B) Compound 15.

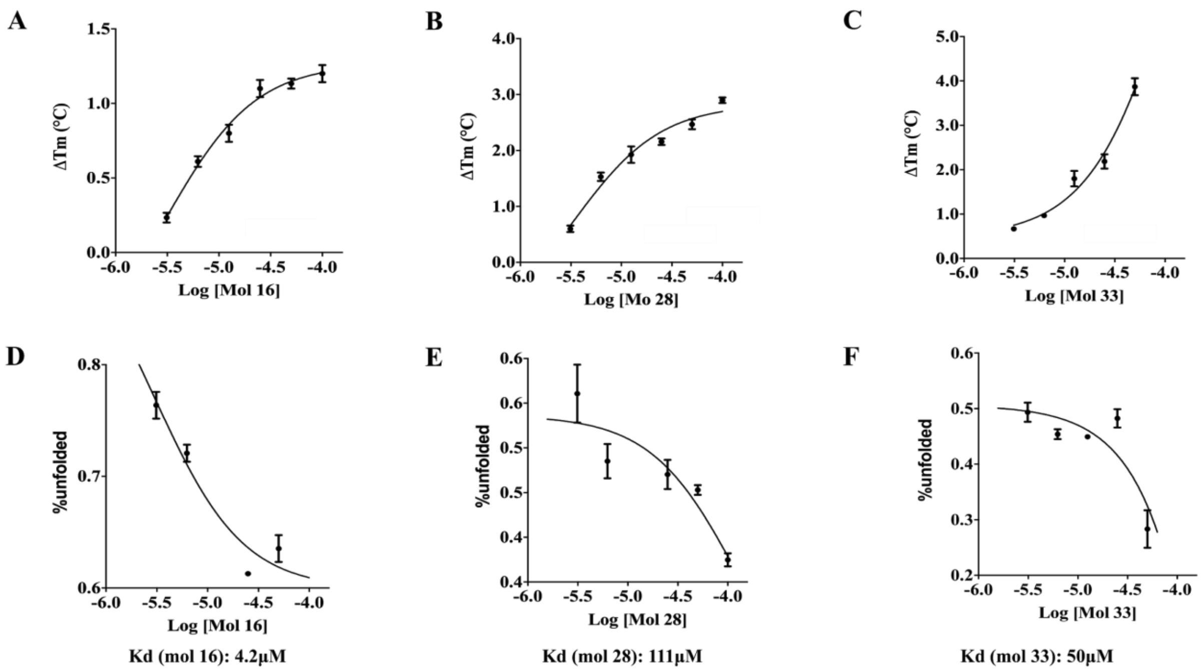

Figure 3S. Effect of different concentrations of compounds **16**, **28** and **33** over the *Pa*PhzS Tm values (upper panel – A-C) and over the fraction on unfolded PaPhzS (lower panel –D-F) at 43˚C. ΔTm was calculated in comparison to DMSO (control) and Kd values were calculated using DSFit software. Plots were generated with GraphPad Prism v.8.0. All data represent the mean ± SD from three independent experiments.
